# Towards improved particle reconstruction for single-molecule localization microscopy using geometric deep learning

**DOI:** 10.1093/bioadv/vbag228

**Published:** 2026-08-11

**Authors:** Diana Mindroc-Filimon, Dominic A Helmerich, Patrick Salome, Markus Sauer, Philip Kollmannsberger

**Affiliations:** Department of Biotechnology and Biophysics, Biocenter, University of Würzburg, Würzburg, Germany; Graduate School of Life Sciences, Würzburg, Germany; Department of Biotechnology and Biophysics, Biocenter, University of Würzburg, Würzburg, Germany; Department of Radiation Oncology, Massachusetts General Hospital, Harvard Medical School, Boston, MA, United States; Translational Radiation Oncology and Medical Physics in Radiation Oncology, German Cancer Research Center, Heidelberg, Germany; Department of Biotechnology and Biophysics, Biocenter, University of Würzburg, Würzburg, Germany; Rudolf Virchow Center for Integrative and Translational Bioimaging, University of Würzburg, Würzburg, Germany; Department of Biotechnology and Biophysics, Biocenter, University of Würzburg, Würzburg, Germany

## Abstract

**Motivation:**

Single-molecule localization microscopy (SMLM) can resolve intracellular structures down to the nanoscale, but often produces sparse and incomplete data. Particle averaging (PA) can aid with the reconstruction of complete structures, but traditional PA methods can suffer from template bias or the high computational costs of geometric alignment.

**Results:**

To address these limitations, we developed a geometric deep learning (GDL) framework for enhanced, template-free 3D particle averaging. Our pipeline uses a GDL autoencoder, trained on high-fidelity simulated data, to map incomplete point clouds into a robust latent space. By averaging feature vectors directly within this space, our method bypasses the need for explicit 3D alignment. We validated our approach on simulated DNA origami and experimental nuclear pore complex (NPC) data. The latent space averaging successfully reconstructed NPC structures with key metrics (ring radius ≈46nm, ring distance ≈52nm) that are comparable to state-of-the-art methods. This work establishes a viable GDL pipeline for SMLM analysis, offering an efficient alternative to traditional PA. While the current model requires structure-specific training, our results highlight the significant potential of GDL for quantitative structural biology.

**Availability:**

https://github.com/dianamindroc/smlm

## 1 Introduction

The emergence of super-resolution microscopy, in particular single-molecule localization microscopy (SMLM), has reshaped cell biology by enabling the visualization of subcellular structures at nanoscale resolution (Sauer and Heilemann 2017, Schermelleh *et al*. 2019). SMLM techniques can localize individual fluorophores with a precision of a few nanometers, yielding reconstructed images with an effective spatial resolution on the order of 20 nm. This capability provides unprecedented opportunities for the quantitative analysis of protein distributions and the structural characterization of molecular complexes *in situ*, with demonstrated applications in neurodegenerative disease research and oncology (Leterrier *et al*. 2017, Nerreter *et al.* 2019).

However, these super-resolution methods are fundamentally limited by the inherent incompleteness of the acquired data. The stochastic nature of fluorophore activation and detection, combined with challenges in achieving dense and uniform labeling, often results in sparse and fragmented representations of the underlying biological structures (van de Linde et al. 2011, Mendes *et al.* 2022). This undersampling hinders the complete reconstruction of molecular architectures and complicates quantitative analysis.

To overcome this challenge, particle averaging (PA) has been widely adopted as a computational strategy to enhance the signal-to-noise ratio and structural completeness (Heydarian *et al*. 2018). Originating from cryo-electron microscopy (Frank 2006), PA combines multiple observations of the same particle, which are often noisy or incomplete, to generate a high-fidelity average. However, conventional PA methods for SMLM data have significant drawbacks. Many PA alogrithms are image-based, requiring the conversion of 3D point cloud data—the direct output of SMLM methods—into a gridded format. This transformation can introduce discretization artifacts and a loss of precision (Löschberger *et al*. 2012). Furthermore, template-based alignment methods risk biasing the final reconstruction towards the chosen template, potentially obscuring novel or heterogeneous structural features (Heydarian *et al*. 2021a).

The intrinsic format of SMLM data as 3D point clouds motivates a shift towards computational tools designed for such non-Euclidean data. Geometric deep learning has recently emerged as a powerful paradigm for analyzing and interpreting complex geometric structures (Bronstein *et al.* 2017). Architectures like PointNet (Qi *et al.* 2017) and its successors can operate directly on point clouds, respecting their permutation invariance and spatial nature. This opens a promising avenue for developing template-free and alignment-agnostic particle averaging workflows. However, the application of geometric deep learning to SMLM data is still incipient and faces challenges, including the lack of ground-truth data to train supervised models and the need to adapt existing architectures to the sparse and noisy nature of biological datasets (Nabi *et al.* 2024).

In this work, we propose a novel framework that leverages geometric deep learning to enhance 3D particle averaging for SMLM, with the aim of reconstructing complete structures from underlabeled or partial observations. Our approach circumvents the need for explicit 3D alignment by encoding particles into a rotation-invariant latent space. We hypothesize that these learned representations can be used to effectively classify heterogeneous structures and that averaging them can robustly reconstruct a complete particle. To enable model training, we first developed a simulation framework to generate high-fidelity SMLM datasets. Our contributions are twofold: we (1) present a method for learning compact and descriptive latent representations directly from the sparse point clouds characteristic of SMLM data, enabling accurate structural classification; and we (2) introduce a particle averaging pipeline that operates within this latent space, thereby eliminating template bias and the computationally intensive alignment step. We demonstrate that this approach can faithfully reconstruct complete biological structures from multiple incomplete instances, offering a significant advancement over state-of-the-art methods. A graphical abstract of the workflow is presented in Fig. 1.

**Figure 1:**
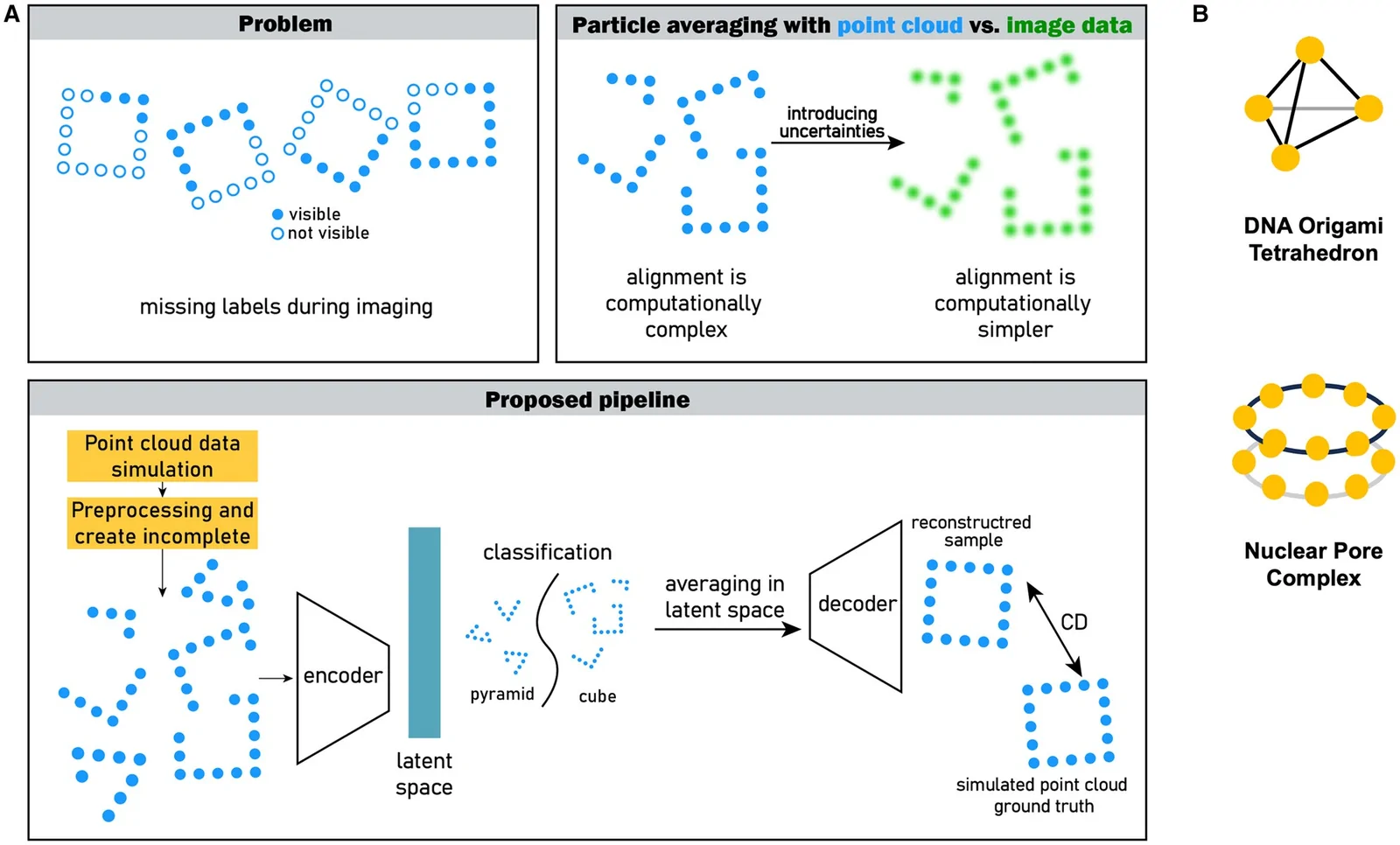
Proposed pipeline. (A) Transforming point cloud data into image data introduces additional uncertainties, motivating our choice of working directly with point cloud data. Typically, alignment of point cloud data is more computationally complex than the alignment of image data. Therefore we decided to work with permutation invariant networks. After data simulation, preprocessing and creating incomplete samples, the point cloud deep learning network is trained for reconstruction and classification in latent space simultaneously. Multiple encoded incomplete samples are averaged and then decoded for obtaining a complete, higher-resolution structure. CD: Chamfer Distance. Encoder and decoder components are detailed in later sections. (B) Illustration of the reference structures used in this study.

## 2 Background and related work

Many particle averaging approaches are template-based, aligning either rendered images (Löschberger *et al*. 2012, Szymborska *et al.* 2013) or coordinate data (Broeken *et al*. 2015) to a predefined structural model, which risks introducing bias. To address this, state-of-the-art methods have shifted towards template-free, coordinate-based particle fusion (Heydarian *et al*. 2018). These techniques perform iterative registration of multiple point clouds, often using Gaussian Mixture Models, to determine particle poses before averaging (Heydarian *et al*. 2021a, Wang et al. 2022). While powerful, these registration-centric methods can be computationally intensive and sensitive to initialization. In parallel, deep learning has been applied to SMLM, but predominantly for image-based tasks like emitter localization from raw camera frames (Speiser *et al*. 2021), not for particle averaging of reconstructed point clouds. The direct application of geometric deep learning to SMLM point clouds for structural analysis remains an emerging field. While pioneering work has used PointNet-based architectures for the specific sub-task of particle alignment (Fan *et al*. 2021), the potential of more advanced models to create an end-to-end particle averaging framework is largely unexplored. To our knowledge this work presents the only application of point cloud deep learning to SMLM particle averaging. Reconstructing a point cloud from its latent space representation presents unique challenges not found in image-based decoding, primarily due to the unstructured and variable-cardinality nature of point set data. A prominent architecture, FoldingNet (Yang *et al.* 2018), addresses this by generating a 3D point cloud by learning a continuous mapping that “folds” a canonical 2D grid into the target shape, guided by the latent vector. While successfully applied to model cell morphology (Vries *et al.* 2022), its efficacy is limited for structures with a non-planar topology. A more versatile approach, particularly for the complex assemblies found in SMLM, is the decoder from the Point Completion Network (PCN) (Yuan *et al.* 2018). This architecture employs a two-stage, coarse-to-fine strategy: it first predicts a sparse point cloud representing the global structural skeleton and then generates dense, high-fidelity details by applying localized folding operations around these initial points. This hierarchical process is adept at capturing both the overall topology and the intricate local features of a structure.

The training of such decoders is guided by loss functions that measure the similarity between the generated and ground-truth point clouds. In the deep learning context, the dominant loss function is the Chamfer Distance (CD), which efficiently computes a symmetric, average distance between each point in one set and its nearest neighbor in the other. This optimization target stands in contrast to the metrics used in traditional, registration-centric particle averaging. In image-based alignment, the objective is typically to maximize a cross-correlation score in pixel space. In coordinate-based alignment, the goal is to minimize spatial distances, either to a fixed template using simple closest-point measures (Löschberger *et al*. 2012) or between pairs of particles. The latter often involves more sophisticated metrics that treat the particles as probability distributions, such as the Bhattacharyya distance between Gaussian Mixture Model representations, to achieve robust, template-free alignment (Heydarian *et al*. 2018).

## 3 Materials and methods

### 3.1 Experimental data

We used an experimental public dataset of macromolecular structures imaged with single-molecule localization microscopy, as described by (Heydarian *et al*. 2021b). The dataset comprises two structures: DNA-origami tetrahedrons and nuclear pore complexes (NPCs). The DNA-origami dataset contains 498 tetrahedron-shaped nanostructures imaged using 3D astigmatic DNA-PAINT. The nanostructures were synthesized via a one-pot reaction with 10 nM scaffold strand (p8064), 100 nM each of core, connector, vertex, biotin, and DNA-PAINT handle staples, and 1400 nM biotin anti-handles. For imaging, 30 000 frames were acquired at an 800 ms integration time using a 2 nM P1-Cy3b imager, with an excitation intensity of 226 W/cm^2^ at 561 nm. The NPC dataset was generated by imaging the Nup107 protein labeled with Alexa Fluor 647 via a SNAP-tag. It includes two subsets, one acquired with 3D astigmatic DNA-PAINT containing 306 particles with an average of 88 localizations per particle and one with 4Pi-STORM containing 750 particles with an average of 58 localizations per particle.

### 3.2 Training data

The training dataset includes 1000 samples of DNA origami tetrahedrons, each consisting of a set $X=\{{\vec{x}}_{1},{\vec{x}}_{2},\ldots ,{\vec{x}}_{k}\}$ of *k* point coordinates $\vec{x}\in R^{3}$. Leveraging statistical parameters extracted from an experimental dataset of 498 DNA-origami tetrahedra (Heydarian *et al*. 2021b), we established probability distributions for key structural features, including the number of localizations per cluster and their standard deviations in three dimensions. This framework simulates predefined geometric scaffolds (e.g. tetrahedra) and populates them with point clouds, incorporating experimentally-derived statistics to enhance biological realism. To address reconstruction challenges, both anisotropic and isotropic point cloud samples were generated by sampling from fitted statistical distributions of the standard deviations. To ensure the simulation framework accurately replicated the statistical properties of real SMLM data, probability distributions were fitted to main experimental metrics extracted from the experimental dataset, specifically the number of localizations per structure, cluster sizes (localizations per binding site), and cluster standard deviations in x, y, and z. Several candidate distributions, including Lognormal, Gamma, and Weibull, were evaluated for goodness-of-fit using the Akaike Information Criterion (AIC). The fidelity of the simulated data to the experimental data was validated through comparative analysis using histogram overlays and statistical assessments, including the Kolmogorov-Smirnov and Mann-Whitney U tests, ensuring the synthetic data accurately reflects real-world experimental conditions.

A standardized data preparation pipeline was established to ensure consistency across diverse datasets for our deep learning applications. To enhance data quality, we performed denoising using HDBSCAN for its proficiency in handling variable density clusters and noise inherent in SMLM data. To simulate realistic biological scenarios of incomplete labeling, we implemented a strategy involving the random removal of entire point cloud clusters, identified by HDBSCAN, to achieve specific labeling efficiencies (e.g. 75% and 50%).

Additionally, preprocessing included rotations and translations to increase data variability mimicking experimental conditions. We also implemented padding by duplication to standardize input dimensions, facilitating consistent batch processing while preserving data integrity. Figure S2 presents preprocessed data for training.

To support systematic degradation experiments, we also simulated NPC data, utilizing the structural architecture described by Theiss *et al*. (2023) as a reference model. These simulations were performed using the same parameters for labeling density, localization precision, and background noise as mentioned previously for the other synthetic datasets, ensuring a consistent and comparable training environment. By generating these high-fidelity NPC configurations with explicit eight-fold rotational symmetry and dual-ring scaffold, we were able to conduct controlled experiments to quantify how well the framework recovers the full canonical geometry from increasingly sparse or incomplete NPC point clouds.

### 3.3 Training loss

To evaluate the reconstruction quality, we use the widely established Chamfer Distance (CD) as a loss function to measure the distance between the reconstructed point cloud and the ground truth. The CD is a measure of the distance between two point sets *A* and *B*, calculated as the average distance between points in *A* to the closest point in *B* and vice versa:


$$d_{\text{Chamfer}}(A,B)=\frac{1}{\left|A\right|}\sum_{a\in A}\underset{b\in B}{\text{min}}\|a-b\|+\frac{1}{\left|B\right|}\sum_{b\in B}\underset{a\in A}{\text{min}}\|b-a\|$$

### 3.4 Training details

The model was implemented using PyTorch and trained on an Nvidia Quadro RTX A5000 GPU with 24GB of memory. We used a 70/20/10 split to divide our dataset into training, validation, and test sets. The model was trained using the Adam optimizer with an initial learning rate of 0.001 and a batch size of 64. The momentum was set to 0.9, and momentum 2 to 0.999. The training was carried out for a maximum of 500 epochs, with an early stopping patience of 30 epochs. A ReduceLROnPlateau scheduler was employed, which reduced the learning rate by a factor of 0.75 if the validation loss did not improve for 10 consecutive epochs. The progression of the training and validation loss throughout this optimization process, demonstrating stable convergence without late-stage overfitting, is provided in Fig. S6. All code for data simulation, network training and validation is freely available under https://github.com/dianamindroc/smlm.

## 4 Results

### 4.1 Data simulation

The dataset used in this work consists of simulated DNA-origami data. DNA origami involves the folding of DNA to create 2D and 3D objects at the nanoscale (Dey *et al*. 2021). Its predictable and programmable nature ensures that the resulting structures are consistent and reproducible, thus they provide an excellent source of ground truth data for the development and evaluation of computational models. A custom data simulation framework was developed to generate realistic synthetic datasets for model training and evaluation. The Lognormal distribution consistently yielded the lowest AIC values across all metrics, identifying it as the optimal model for generating realistic synthetic data. The Kolmogorov-Smirnov (KS) test confirmed no statistically significant difference between the datasets (P>0.05), thereby verifying the simulation’s suitability for supervised network training.

Samples of simulated tetrahedron are presented in Fig. S1, displaying structural fidelity to the experimental data in Heydarian *et al*. (2021a). Analysis of the reconstructed particles yielded a mean edge length of 88.57 ± 8.21 nm (95% CI: [86.94, 90.20]) and a mean radius of 57.70 ± 4.78 nm (95% CI: [56.75, 58.65]). While the original dataset analysis primarily focused on resolving rotational symmetries, our extracted dimensions align well with the known 100 nm programmable design geometry of the DNA origami scaffold.

### 4.2 Model selection

To identify the optimal encoding architecture for SMLM data, a comparative analysis of five leading neural network models was performed. We evaluated AtlasNet (Groueix *et al.* 2018), FoldingNet (Yang *et al.* 2018), Point Completion Network (PCN) (Yuan *et al.* 2018), PointNetFCAE (Du *et al*. 2023), and TopNet (Tchapmi *et al.* 2019) based on reconstruction fidelity, computational performance, and pose-invariance. Each model was initially trained on the large-scale ShapeNet benchmark dataset as intended by their original design. Subsequently, the pre-trained models were tasked with completing partial structures from our experimental DNA-origami dataset. The primary evaluation criterion was reconstruction accuracy, which was quantified using the Chamfer Distance between the generated output and the ground truth point cloud.

The results of the comparison study for choosing the point cloud deep learning architecture that works best on SMLM data are reported as CD across the test set and presented in Table 1.

**Table 1:** Results of the comparison study of different state-of-the-art point cloud deep learning architectures on DNA origami.

Architecture	Chamfer distance
FoldingNet	1.98 [1.79–2.11]
**PCN**	**0.16 [0.12–0.18]**
TopNet	8.37 [7.98–8.88]
AtlasNet	7.85 [7.21–8.03]
PointNetFCAE	7.99 [7.42–8.23]

The reported measure is the Chamfer Distance between reconstruction and ground truth for the point completion task with 95% confidence interval based on *t*-distribution.

Bold value represents the best performing architecture.

From the models evaluated, the Point Completion Network (PCN) was selected as starting point for further refinement.

### 4.3 Point cloud deep learning for SMLM

We developed Pocafoldas, a network tailored for the reconstruction and analysis of SMLM point cloud data. The network’s encoder is founded on a hierarchical feature learning strategy inspired by PCN Yuan *et al*. (2018). It first processes the input point cloud with a convolutional network to extract point-wise features, which are then aggregated via max-pooling to form a global feature descriptor. This global descriptor is concatenated back with the initial point-wise features to create a rich hybrid representation that preserves both local geometric detail and global context. This representation is processed by a self-attention mechanism, which adaptively refines the feature space by emphasizing structurally important points. The final 1024-dimensional latent vector is generated using mean-pooling, a deliberate choice over max-pooling to create a global descriptor that better reflects the overall distribution and density of the localizations.

The decoder implements a two-stage, coarse-to-fine reconstruction process. It first uses a multi-layer perceptron (MLP) to transform the global feature vector into a coarse structural scaffold. This scaffold is then refined using an adaptive folding mechanism, where a canonical 2D grid is deformed into a detailed 3D structure based on the learned global features. We modified the decoder to dynamically adjust the size of the output point cloud to match that of the input. Unlike standard models with fixed outputs, it preserves the quantitative nature of the localizations and prevents the network from hallucinating points not present in the experimental data. The architecture and data flow pipeline are visualized in Fig. 2. More architectural details are presented in the Appendix, available at supplementary material  *Bioinformatics Advances* online.

**Figure 2:**
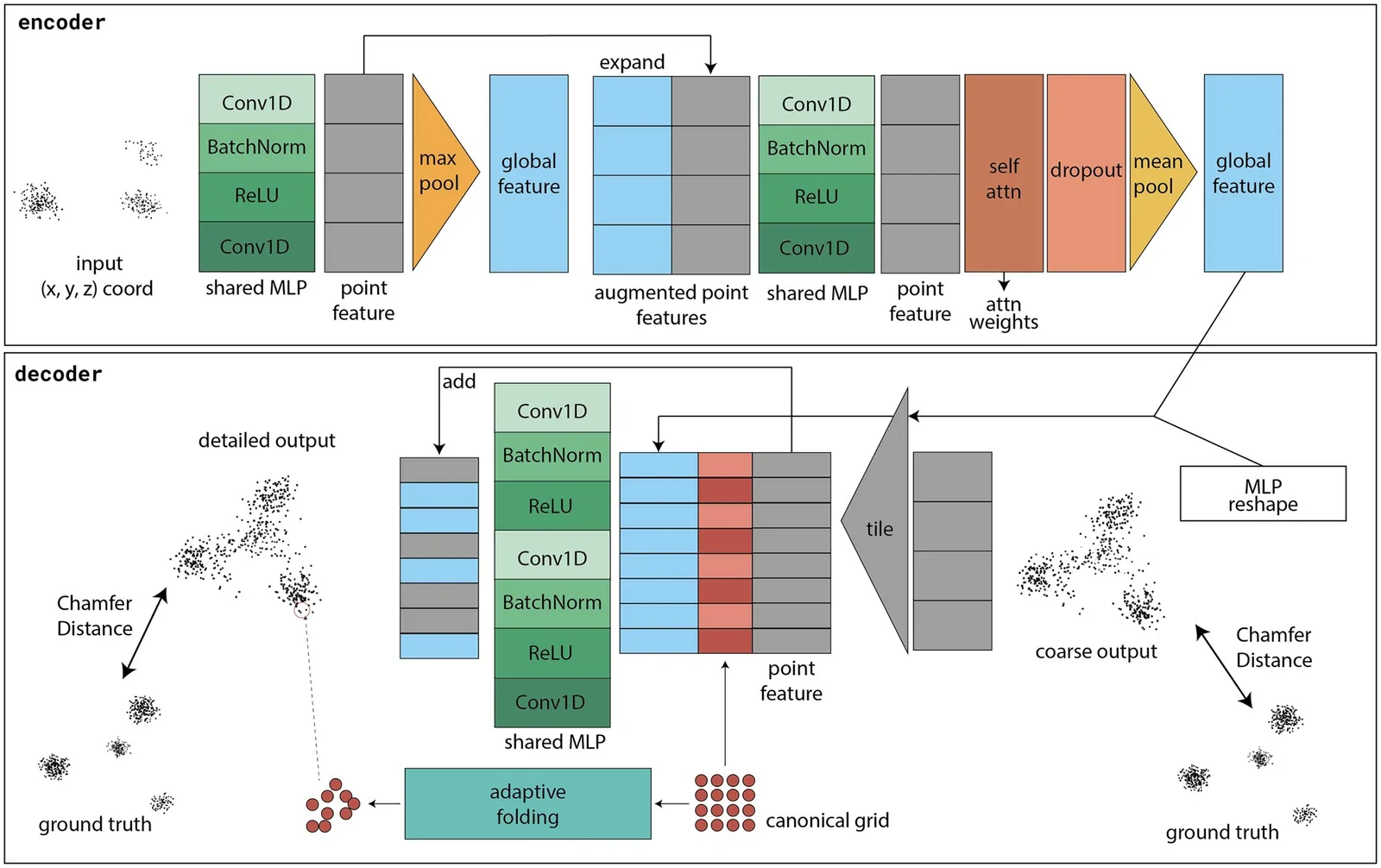
Architecture of the Pocafoldas network. The model processes input point clouds through a series of shared MLPs and pooling operations to extract global features. We introduced key improvements including dropout and self-attention mechanisms to enhance feature learning and model generalization. The network produces both coarse and detailed outputs, with the latter refined through an improved folding operation, called adaptive folding. This operation allows for more accurate reconstruction of complex geometries. Both outputs are compared against ground truth data using Chamfer distance as a metric.

### 4.4 Clustering in latent space

To investigate the quality of the learned representations, we visualized the 1024-dimensional latent space of encoded simulated DNA-origami cubes and tetrahedra using t-Distributed Stochastic Neighbor Embedding (t-SNE) van der Maaten and Hinton (2008). To test the robustness of the encoding against incomplete data, we generated t-SNE projections for structures with 0, 1, and 2 clusters systematically removed.

Our encoding strategy successfully generates a robust latent representation that distinguishes between different geometries while remaining invariant to missing data. As illustrated in the t-SNE projection (Fig. S5), the encoded cube and tetrahedron structures occupy distinct, well-separated regions of the latent space. This separation persists even after the removal of one or two clusters—within each class, the representations of complete and incomplete structures cluster together, confirming that the model effectively recognizes them as variants of the same object.

### 4.5 Systematic experimentation

We conducted a systematic evaluation of the network architecture through two distinct training approaches: autoencoding and point cloud completion.

The initial autoencoder training was designed to assess the model’s ability to create high-fidelity reconstructions of its own input and to generate a meaningful latent space. In this phase, we challenged the network with various data types, including simulated point clouds with both isotropic and anisotropic distributions, multiple sample sizes, and distinct geometries (tetrahedra and cubes).

In the subsequent point cloud completion training, the network was tasked with a more complex objective: reconstructing complete, isotropic ground truth structures from incomplete and anisotropic inputs. To identify the most effective architecture, we performed a systematic, incremental evaluation of key components. We began with a baseline model and sequentially integrated an adaptive folding mechanism, a self-attention module, and a dropout layer. An ablation study was also conducted by removing the folding mechanism entirely to assess the network’s capacity for direct point generation. The optimal model configuration was first determined through rigorous training on our simulated DNA-origami dataset. We then applied the adapted network on simulated incomplete underlabelled DNA origami data with anisotropic noise. An example of the DNA origami tetrahedron from the reconstruction results on the test set with the best performance can be seen in Fig. 3, with CDs of 0.2521, 0.2375 and 0.2267 respectively. To further characterize reconstruction fidelity, we computed spatial error maps. These maps reveal that while the network accurately reconstructs dense cluster centers, higher spatial variability naturally occurs at the sparser peripheral regions of the point clouds (Fig. S8). This best-performing architecture was then subsequently trained and evaluated on the experimental NPC dataset using the pretrained checkpoint. Completion results are presented in Fig. 4. While the model successfully reconstructs the majority of these experimental particles, an analysis of feature-space outliers revealed that highly sparse or irregular input localizations can lead to reduced axial fidelity. In these specific cases, the model accurately captures the coarse lateral ring-like organization, but fails to clearly resolve the axial separation between the cytoplasmic and nucleoplasmic rings (Fig. S7).

**Figure 3:**
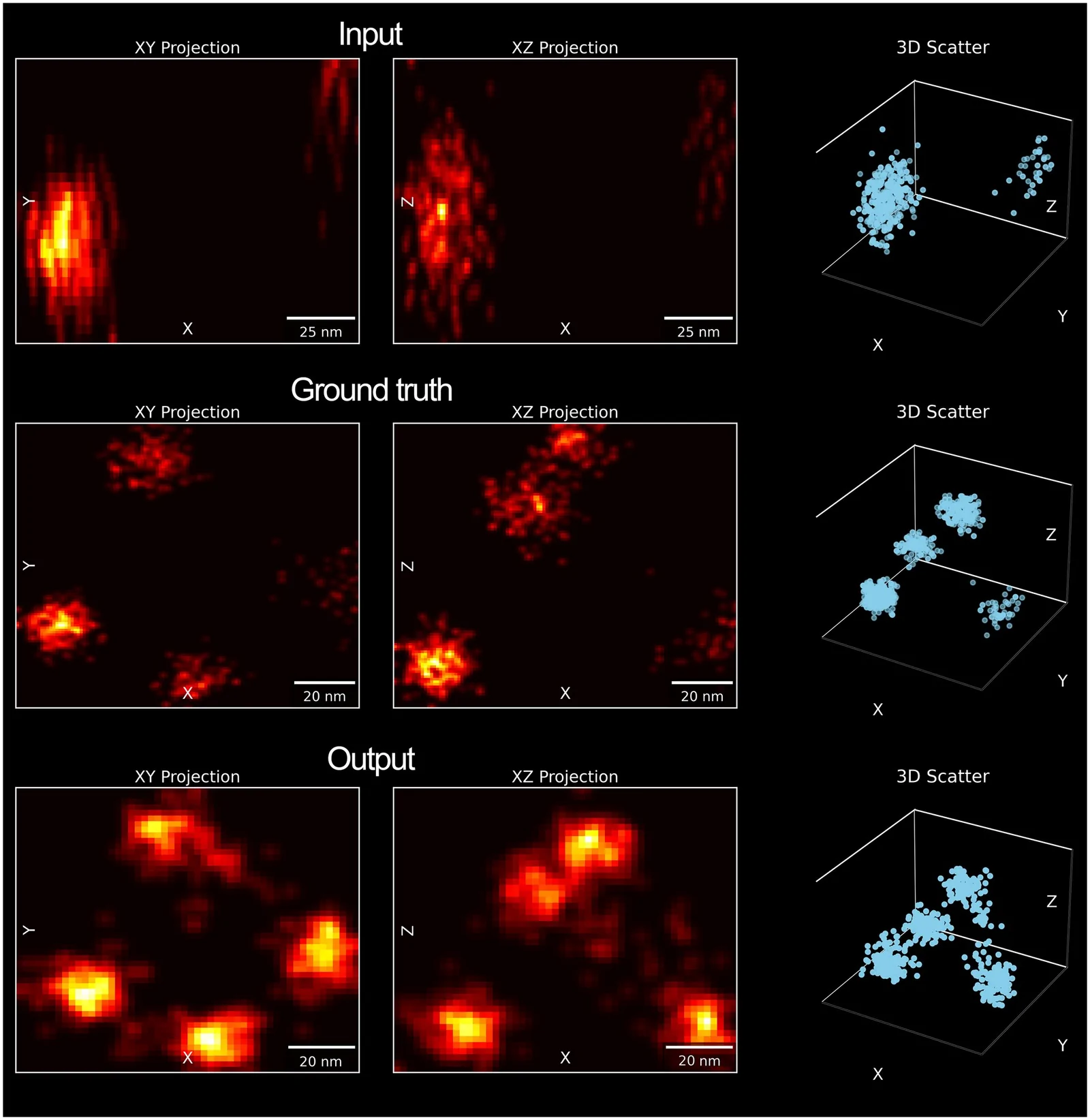
DNA origami completion results. Top: Input sample; middle: Ground truth sample; bottom: Reconstructed sample. *XY*, *XZ* projections and 3D scatter. The reconstructed output shows high similarity to the ground truth, even with highly incomplete and distorted input data, accurately restoring the 3D positions and morphology of the DNA-origami assemblies, and highlighting the model’s ability to perform high-fidelity structural completion.

**Figure 4:**
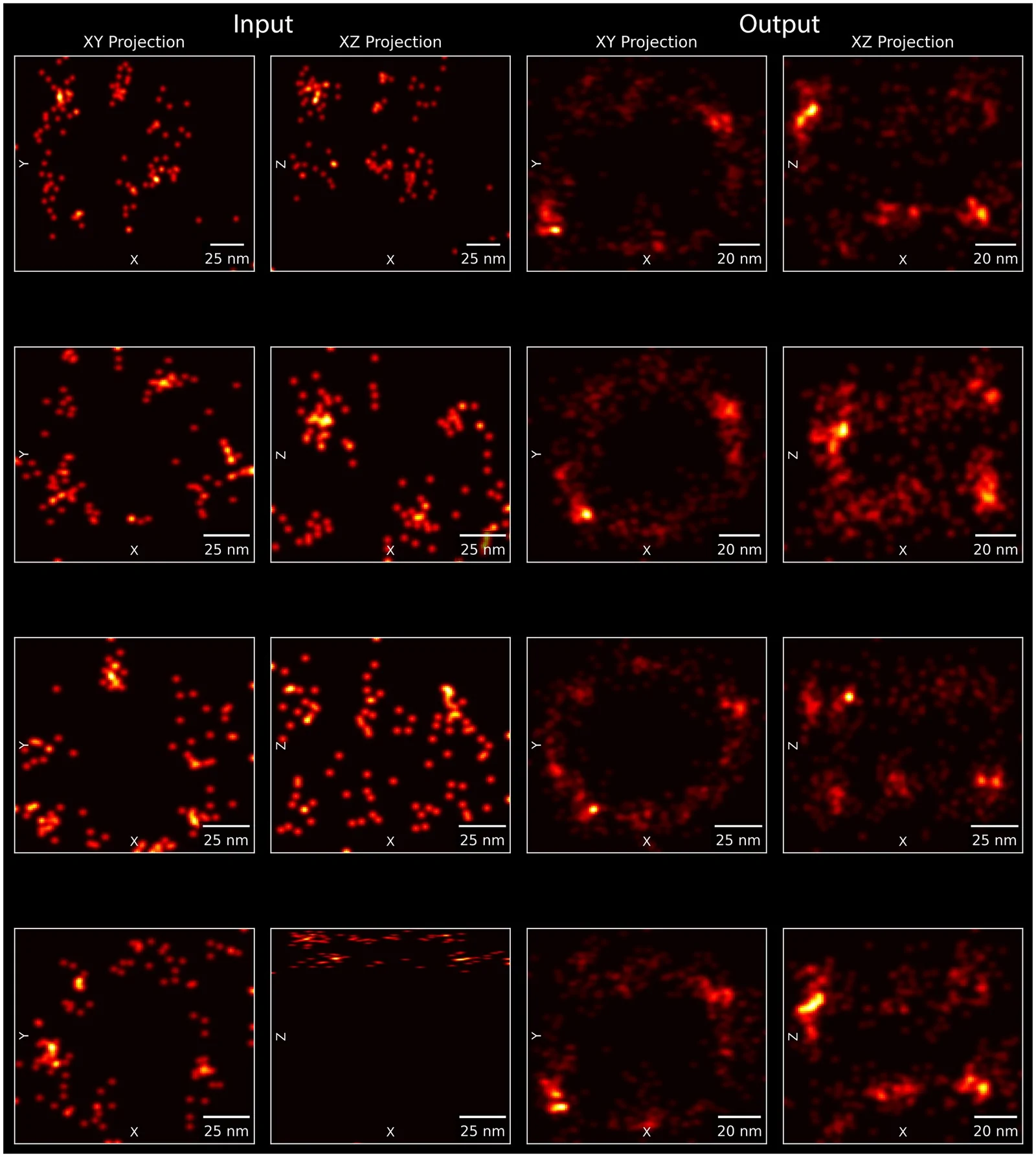
Nuclear pore complex reconstruction results. Left: input point clouds, *XY* and *XZ* projections. Right: reconstructed point clouds, *XY* and *XZ* projections.

### 4.6 Towards enhanced particle averaging

Following encoding, the SMLM point clouds are averaged in latent space. This process leverages the permutation-invariant nature of the encoded representations, circumventing the challenge of establishing point-to-point correspondences required by traditional averaging methods. Rather than decoding the direct mean of the latent vectors, we identify a medoid: the single feature vector from the original dataset closest to the average representation, as determined by the squared Euclidean distance. While a medoid is canonically defined as the point that minimizes the sum of pairwise distances to all other points in a cluster, our approach of minimizing the squared distance to the mathematical centroid is strictly algebraically equivalent. This equivalence allows us to identify the true medoid with significantly higher computational efficiency (detailed in Appendix, available at supplementary material  *Bioinformatics Advances* online). This medoid is then decoded to generate the final, averaged structure, ensuring that the resulting point cloud is a high-likelihood representation from the learned data manifold, which mitigates the risk of generating artifacts from decoding a vector that lies outside the distribution of observed structures. Direct mathematical averaging to a centroid can yield out-of-distribution structures with reduced geometric parameters, whereas the medoid successfully preserves the expected biological dimensions (Fig. S10).

As illustrated in Fig. 5, the decoded medoid successfully reconstructs the canonical architecture of the NPC. The model resolves the distinct cytoplasmic and nucleoplasmic rings, forming the well-known double-ring structure. Furthermore, the observed ring spacing and approximate symmetry are consistent with known NPC morphology. To explicitly verify the recovery of this canonical architecture, we analyzed the angular density profiles of the reconstructed particles. This analysis confirmed the data-driven recovery of the eight-fold rotational symmetry across both simulated and experimental datasets, effectively reflecting the inherent biological variance (Fig. S9).

**Figure 5:**
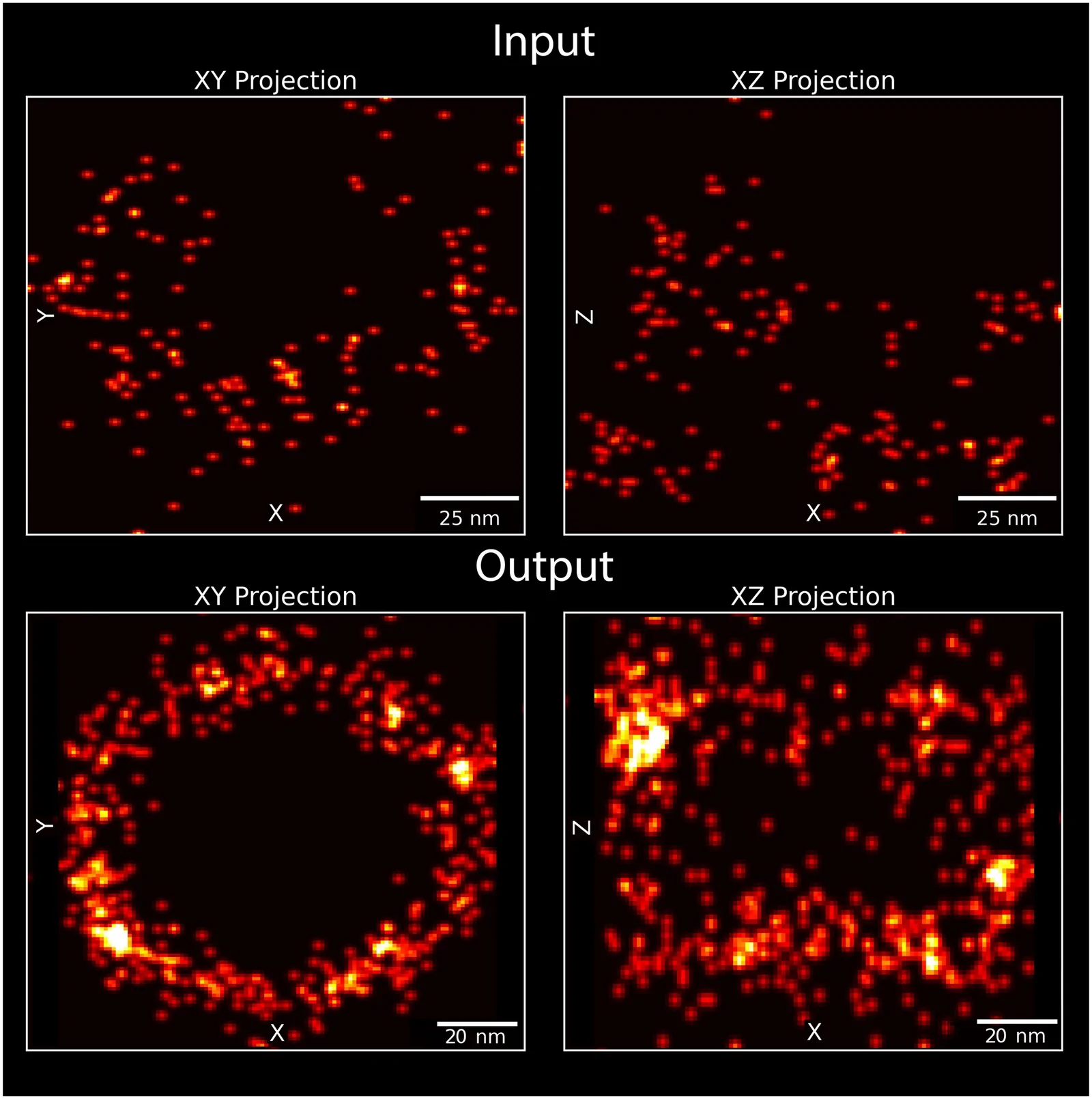
The decoded NPC closest to the centroid, which was calculated by averaging all encoded feature vectors from the test set. Top: input, Bottom: output.

### 4.7 Quantitative comparison

The performance of the methods developed in this work was benchmarked by comparing key structural metrics against template-free all-to-all registration as described in (Heydarian *et al*. 2021a) and cryo-EM measurements. These metrics were extracted from the average consensus particle structure (i.e. the decoded medoid, Fig. 5), which is a single latent. The analysis included characterizing ring diameters from the radial distribution of localized points (Fig. 6A) and determining the position and separation of stacked rings from their axial distribution, as represented by the z-coordinate histogram (Fig. 6B).

**Figure 6:**
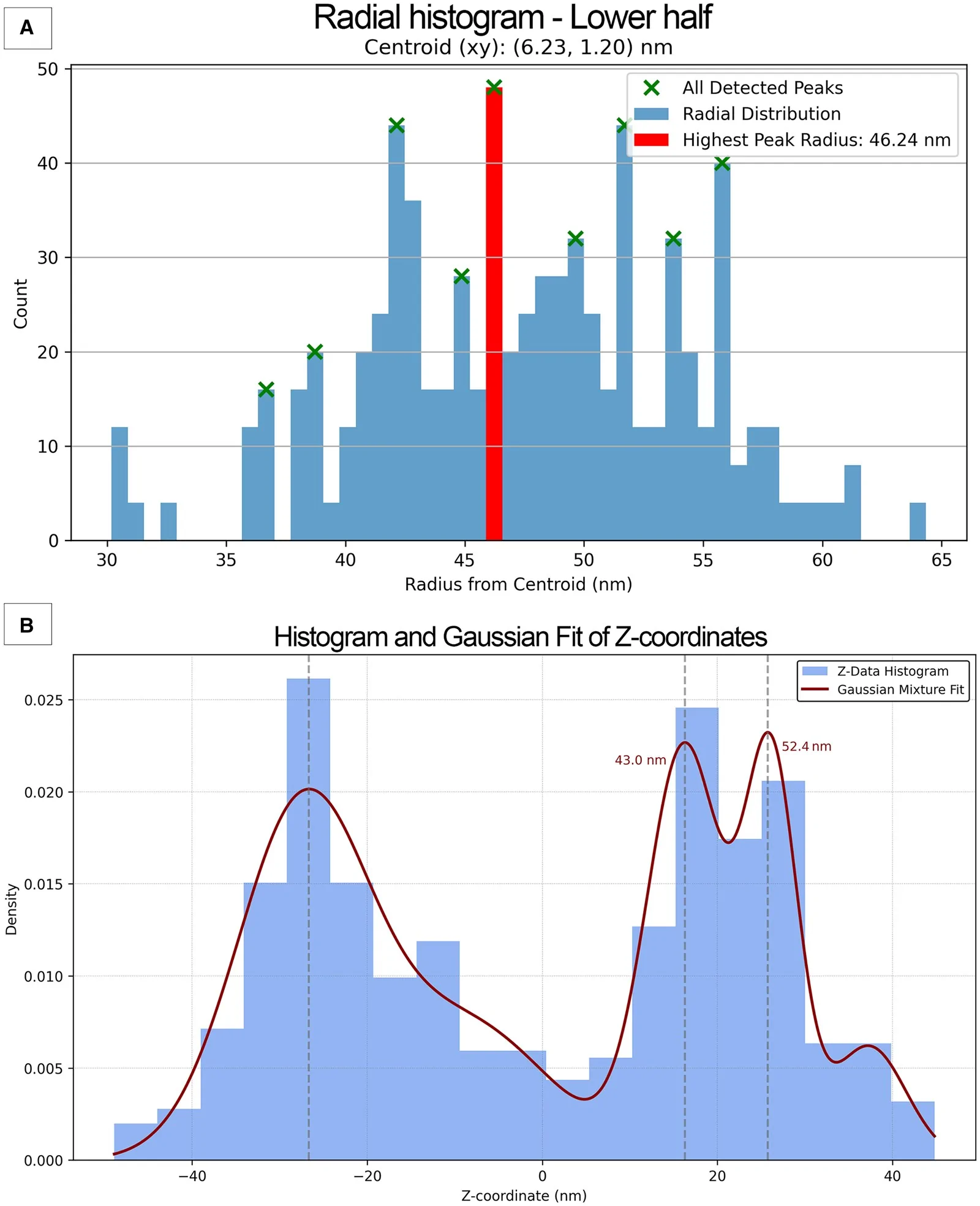
(A) Radial distribution analysis for the nuclear ring of the reconstructed NPC. The histogram shows the count of points as a function of radius from the centroid (6.23, 1.20 nm). Detected peaks are marked with green ‘x’, with the highest peak (red bar) identified at a radius of 46.24 nm. (B) Z-histogram of reconstructed NPC. A Gaussian Mixture Model (GMM) fitted to the distribution reveals a multi-modal profile characteristic of the NPC’s architecture. The GMM mathematically estimates the distance between the primary peaks at 52.4 nm, consistent with the expected spacing between the cytoplasmic and nuclear rings. An intermediate peak (at 43.0 nm separation) is attributed to a local density hotspot within the reconstructed point cloud, likely an artifact.

The radial histogram indicates a nuclear pore complex (NPC) radius of 46 nm (Fig. 6A). The z-histogram reveals a distance of 52.4 nm between the cytoplasmic and nuclear rings (Fig. 6B) for the consensus structure. To quantitatively assess the dimensions and structural variability of the reconstructions, we applied Gaussian fitting to the radial and axial cross-sections of N=56 individually reconstructed particles in the test set. We extracted a mean cytoplasmic ring radius of 46.9 ± 2.9 nm (95% CI: [45.10, 47.29]) and a mean nuclear ring radius of 47.1 ± 2.0 nm (95% CI: [46.03, 47.96]). Furthermore, the axial separation between the cytoplasmic and nuclear rings (inter-ring distance) was measured at 49.1 ± 4.7 nm (95% CI: [47.70, 50.31]).

In Table 2, we compare the metrics derived with our method to those reported by Heydarian et al. Heydarian *et al*. (2021a), who applied a template-free averaging method, and to data from the high-resolution cryo-EM structural map of the human NPC scaffold by von Appen *et al*. (2015).

**Table 2:** Comparison of structural metrics for NUP107.

Metric NUP107 (nm)	Heydarian et al.	cryo-EM (von Appen et al.)	This work
Ring radius	51.8	46.6	46.2
Ring distance	61.6	59.0	52.4

Ring radius is the radius of the nuclear ring and ring distance is the distance between cytoplasmic and nuclear rings.

To establish the operational limits of our framework, we conducted systematic degradation experiments on simulated NPC data. By systematically subjecting ground-truth structures to random localization downsampling and the targeted removal of entire structural subunits, we observed a quantifiable and predictable decline in geometric accuracy and symmetry recovery. Specifically, removing up to three structural subunits caused the inferred ring radius and inter-ring distance to shrink from their baselines (48.8 nm and 59.5 nm) down to 44.9 nm and 55.6 nm, respectively, before plateauing. Similarly, random downsampling revealed that while the network maintains 8-fold symmetry ($\Psi_{8}$) up to a 20–30% loss of localizations, extreme sparsity (70% removal) progressively degrades the symmetry score to 0.3 (Fig. S11).

### 4.8 Computational efficiency

We evaluated the computational efficiency of the model on a held-out test set of 200 samples. The network comprises 2.80 million parameters. The model requires 0.23 GFLOPs per sample, yielding an average latency of 3.78 ms on CPU and 1.54 ms on an Nvidia Quadro RTX A5000 GPU with 24GB of memory.

## 5 Discussion

This work introduces a novel geometric deep learning pipeline to address main challenges in 3D single-molecule localization microscopy particle averaging, namely data incompleteness and the computational cost of traditional alignment. Our innovation is a method that performs particle averaging directly in a learned latent space, bypassing the need for explicit point cloud registration. This approach provides a computationally efficient and robust framework for reconstructing complete structures from sparse, noisy observations.

The main element of our pipeline is the Pocafoldas network, an encoder-decoder architecture specifically tailored for SMLM data. To enable supervised training, we first developed a custom simulation framework that generates realistic point clouds by sampling from statistical distributions derived from experimental DNA origami data. The lack of ground truth information in experimental localization microscopy data makes it challenging to train accurate models. Therefore, using simulations can provide a controlled environment to generate high-quality training data, hence the approach provided annotated datasets with corresponding ground truth. The Pocafoldas architecture was then systematically optimized through key modifications: a self-attention mechanism to focus on geometrically salient features, mean pooling to generate a more holistic global feature descriptor, and a folding-based decoder with dynamic output sizing to reconstruct the structure with a natural point density. These adaptations were aimed at learning meaningful structural representations from the sparse and incomplete point clouds characteristic of SMLM.

Our primary contribution is particle averaging that operates within the permutation-invariant latent space learned by Pocafoldas. Instead of performing iterative geometric alignment on raw coordinates, our method averages the latent space vectors of all particles and identifies the medoid (the latent vector of an observed particle closest to the average). Decoding this medoid vector yields a high-quality consensus structure. This approach is computationally efficient at inference time and inherently robust to input variability. When applied to experimental data of the nuclear pore complex (NPC), our method yielded key structural measurements (mean cytoplasmic ring radius 46.9 ± 2.9 nm, mean nuclear ring radius of 47.1 ± 2.0 nm, inter-ring distance of 47.1 ± 4.7 nm). This low particle-to-particle variability demonstrates that despite the broad localization spread inherent to SMLM data, our geometric deep learning pipeline yields highly stable and reproducible structural parameters that show strong agreement with high-resolution cryo-EM data and other state-of-the-art SMLM analysis methods, validating the effectiveness of this latent-space-centric approach. The broad spatial distribution observed in the radial profiles reflects the inherent physical stochasticity of SMLM acquisitions—including localization uncertainty and natural conformational heterogeneity—rather than algorithmic artifacts. Because our pipeline reconstructs the structure by identifying a representative experimental medoid rather than computing a smoothed mathematical average, this variance is naturally preserved in our final structural estimates.

The main limitation of our current method is its structure-specificity, as the Pocafoldas network must be retrained for each new macromolecular target. Future work should focus on overcoming this limitation to enhance broad applicability, by potentially developing generalizable architectures, incorporating richer per-localization features like photon counts, and leveraging higher-fidelity training data. While the current model is structure-specific, this framework provides a clear path for enhancing the reconstruction of filamentous proteins such as microtubules. By learning the underlying manifold of a continuous filament, the network can map sparse or fragmented experimental localizations to a complete structural representation in latent space, effectively bridging structural gaps caused by variable labeling efficiency or signal intensity, but methods to specifically reconstruct filaments such as Berberich *et al*. (2021) might perform better in this case.

This research successfully demonstrates the viability and potential of a GDL-based pipeline for SMLM structural analysis. By shifting the complex task of particle averaging from coordinate space to a learned, information-rich latent space, we have established an efficient and robust proof-of-concept for analyzing incomplete structural data.

## Supplementary Material

vbag228_Supplementary_Data

## Data Availability

The experimental datasets used in this study, comprising localization data for NUP107 and 3D DNA-origami tetrahedrons, were obtained from the 4TU.ResearchData repository at https://doi.org/10.4121/13797686. A demonstration dataset containing simulated tetrahedron structures is available in the project repository at https://github.com/dianamindroc/smlm. All code for data simulation, network training and validation is freely available under https://github.com/dianamindroc/smlm.
