# Supplementary material for: Towards improved particle reconstruction for single-molecule localization microscopy using geometric deep learning
Source: Bioinform Adv. 2026 Aug 11;6(1):vbag228. doi: 10.1093/bioadv/vbag228 (PMC13619314; doi:10.1093/bioadv/vbag228)
Supplement: Supplementary file 1 [file vbag228_supplementary_data.pdf]

Supplementary Material

Medoid definition

In our pipeline, the medoid is computationally identified as the experimental sample whose latent feature vector minimizes the squared Euclidean distance to the mathematical average (the centroid) of the cluster. While the canonical definition of a medoid is the point that minimizes the sum of distances to all other points in the dataset, our approach is mathematically equivalent under squared Euclidean distance.

Specifically, for a set of N points, minimizing the sum of squared pairwise distances from a candidate point x_i to all other points x_j is algebraically identical to minimizing the distance from x_i to the centroid μ. This can be demonstrated by expanding the sum of squared distances:

$$\sum_{j=1}^{N} \|x_i - x_j\|^2 = \sum_{j=1}^{N} \left(\|x_i\|^2 - 2x_i \cdot x_j + \|x_j\|^2\right) \tag{1}$$

By distributing the summation and substituting the definition of the centroid ($\mu = \frac{1}{N}\sum x_j$), this simplifies to:

$$\sum_{j=1}^{N} \|x_i - x_j\|^2 = N\|x_i - \mu\|^2 + \sum_{j=1}^{N} \|x_j\|^2 - N\|\mu\|^2 \tag{2}$$

Because the terms $\sum \|x_j\|^2$ and $N\|\mu\|^2$ are constants for a given cluster and are entirely independent of the choice of x_i, minimizing the pairwise distance sum is strictly equivalent to minimizing $N\|x_i - \mu\|^2$. We utilized this equivalence to circumvent the $\mathcal{O}(N^2)$ computational complexity required for exhaustive pairwise distance calculations, effectively reducing the medoid search to a highly efficient $\mathcal{O}(N)$ operation without compromising mathematical rigor.

Architectural details

The decoder directly receives the 1024-dimensional global feature vector generated by the encoder. Initially, a multi-layer perceptron transforms this global feature vector into a sparse, coarse structural scaffold. To produce the subsequent detailed output, the network must combine this global context with local geometry. The grey features in the wide part of the "tile" operation represent the global feature vector being replicated, or tiled, so it can be concatenated with the point features of the coarse scaffold. The coarse and detailed outputs serve distinct purposes in the hierarchical coarse-to-fine strategy: the coarse output predicts a sparse point cloud representing the global structural skeleton, whereas the detailed output generates dense, high-fidelity local details by applying localized operations around these initial points. Finally, the red square grid illustrated in the adaptive folding step represents a canonical 2D grid. The adaptive folding mechanism takes this generic 2D grid and deforms it into a detailed 3D structure, guided by the learned features.

The adaptive folding computational process begins by concatenating the global feature vectors, which encode the overall shape characteristics of the particle, with canonical 2D grid coordinates that provide local spatial relationships. This combined input is then fed into a multi-layer perceptron consisting of three fully connected layers, each equipped with Rectified Linear Unit (ReLU) activations. As the concatenated features pass through this multi-layer perceptron, the network progressively refines the representation, effectively transforming it from a high-dimensional latent feature space down to precise three-dimensional Euclidean coordinates. During forward propagation, the module meticulously aligns and expands the feature dimensions to ensure a robust correspondence between the learned global features and the individual grid points. This alignment phase allows the network to mathematically fold the 2D grid around each coarse point into a dense, highly detailed 3D local patch.

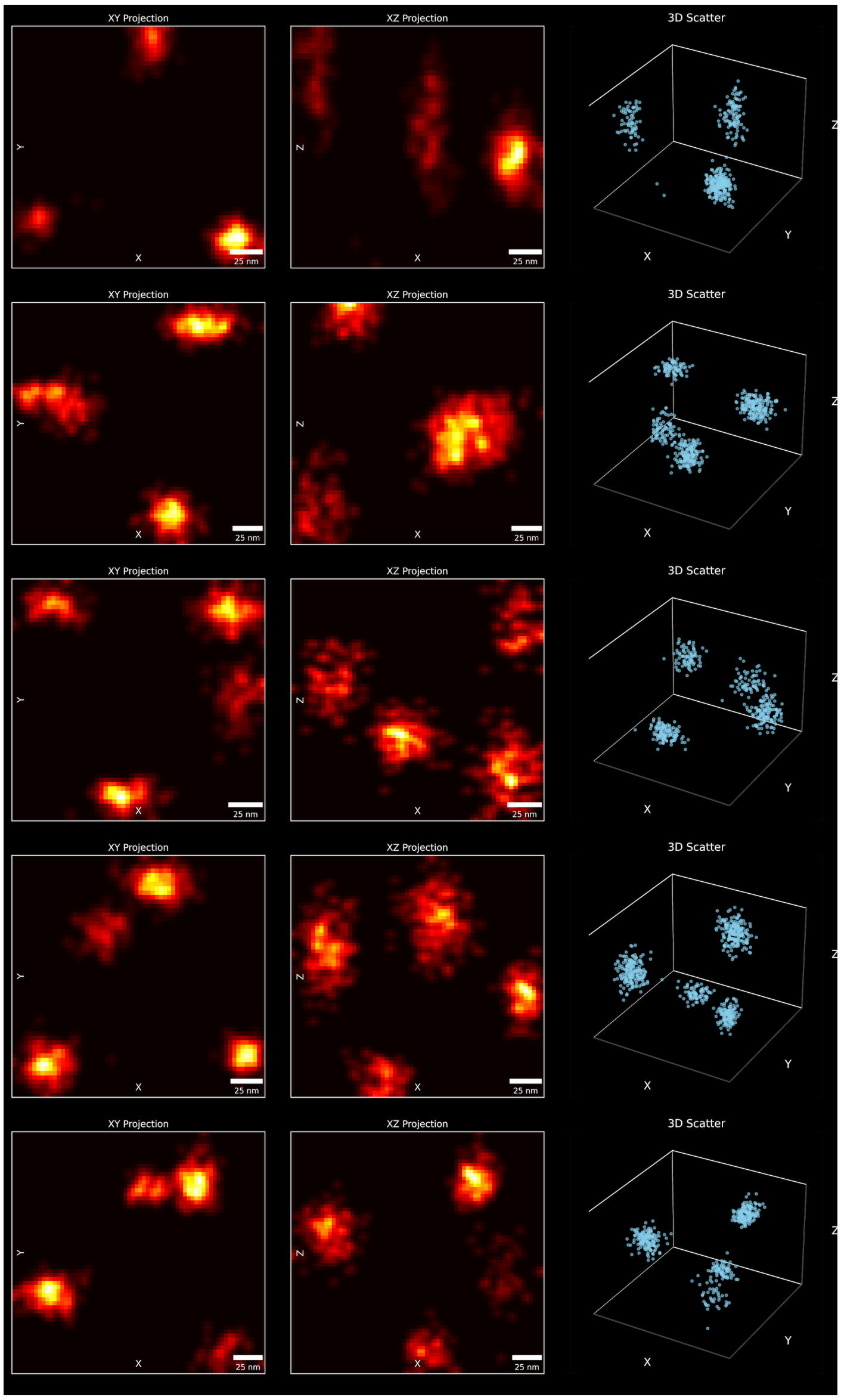

Figure S1 Simulated DNA-origami tetrahedron samples; Left: 2D projections of the simulated tetrahedron point clouds onto the XY and XZ planes, right: 3D scatter plot of the same tetrahedron sample, providing a volumetric view of the localization clusters

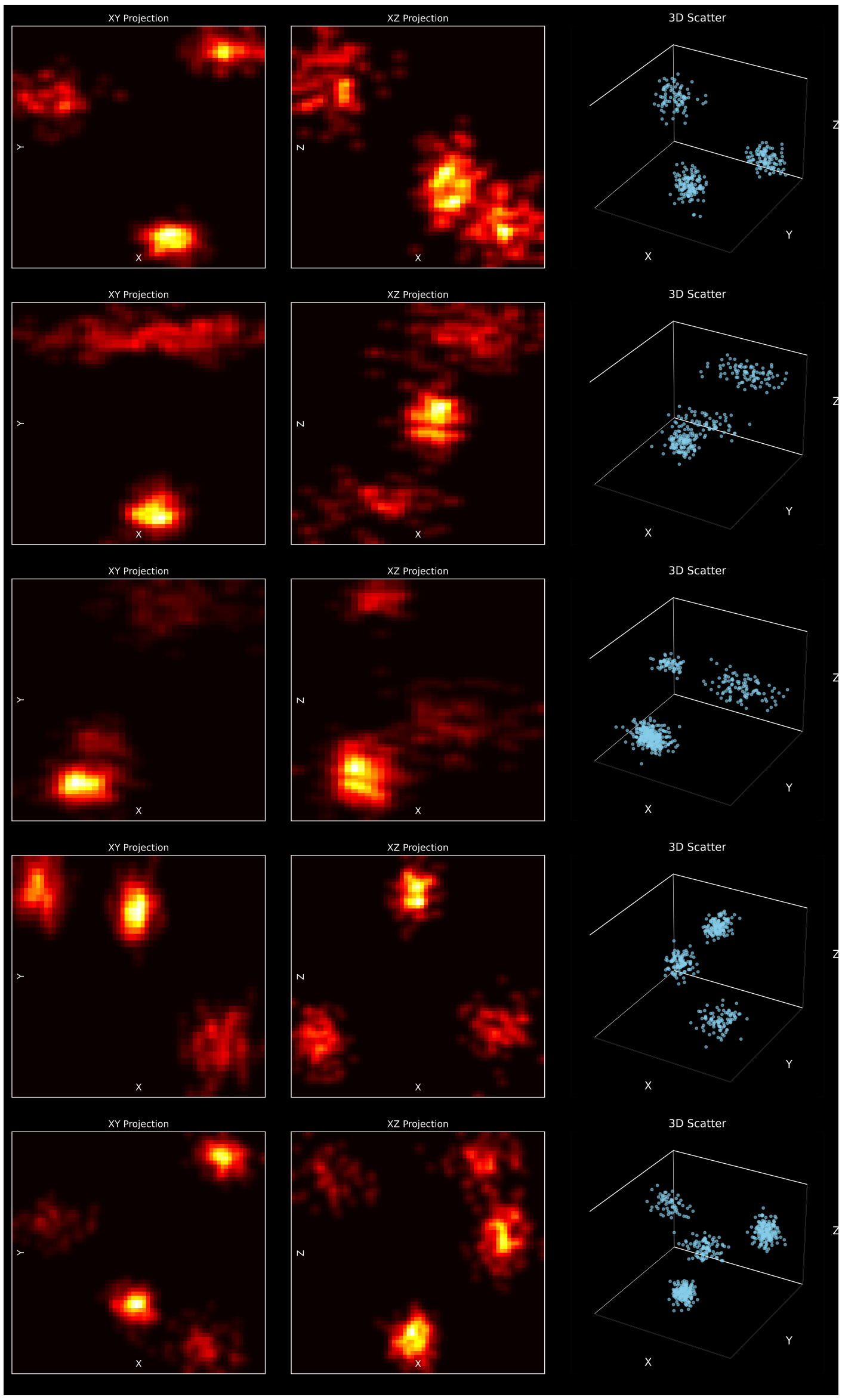

Figure S2 To augment the training dataset and enhance the network's robustness to experimental variability, the preprocessing pipeline incorporated random rotations and translations. Furthermore, to simulate varying labeling efficiencies and imaging conditions, we applied partial cluster removal and introduced anisotropic noise to the point clouds. These augmented data served as the training input for the network. Five distinct samples are presented here as XY, XZ projections and 3D scatter, with missing clusters, added anisotropic noise and rotations.

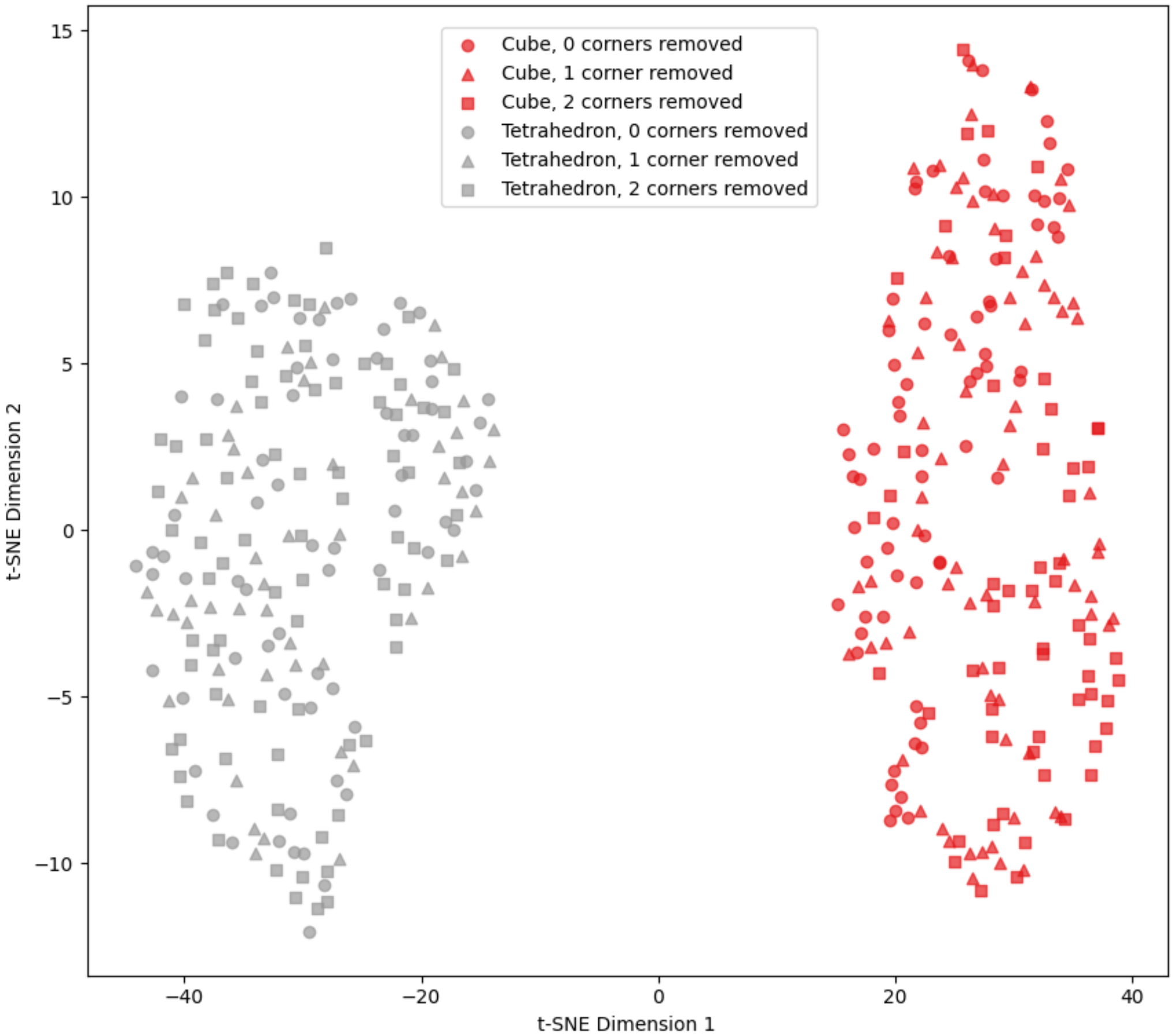

Figure S3 t-SNE visualization of DNA origami structures. The plot shows the distribution of cube (red) and tetrahedron (gray) structures in a two-dimensional space derived from their high-dimensional feature representations. Each point represents a single structure, with different shapes indicating the number of corners removed: circles (0), triangles (1), and squares (2). The clear separation between red and gray clusters demonstrates that the model effectively distinguishes between cubes and tetrahedron. Within each structural class, the overlapping distribution of different shapes suggests that the model is robust to corner removal, treating structures with varying numbers of removed corners as similar.

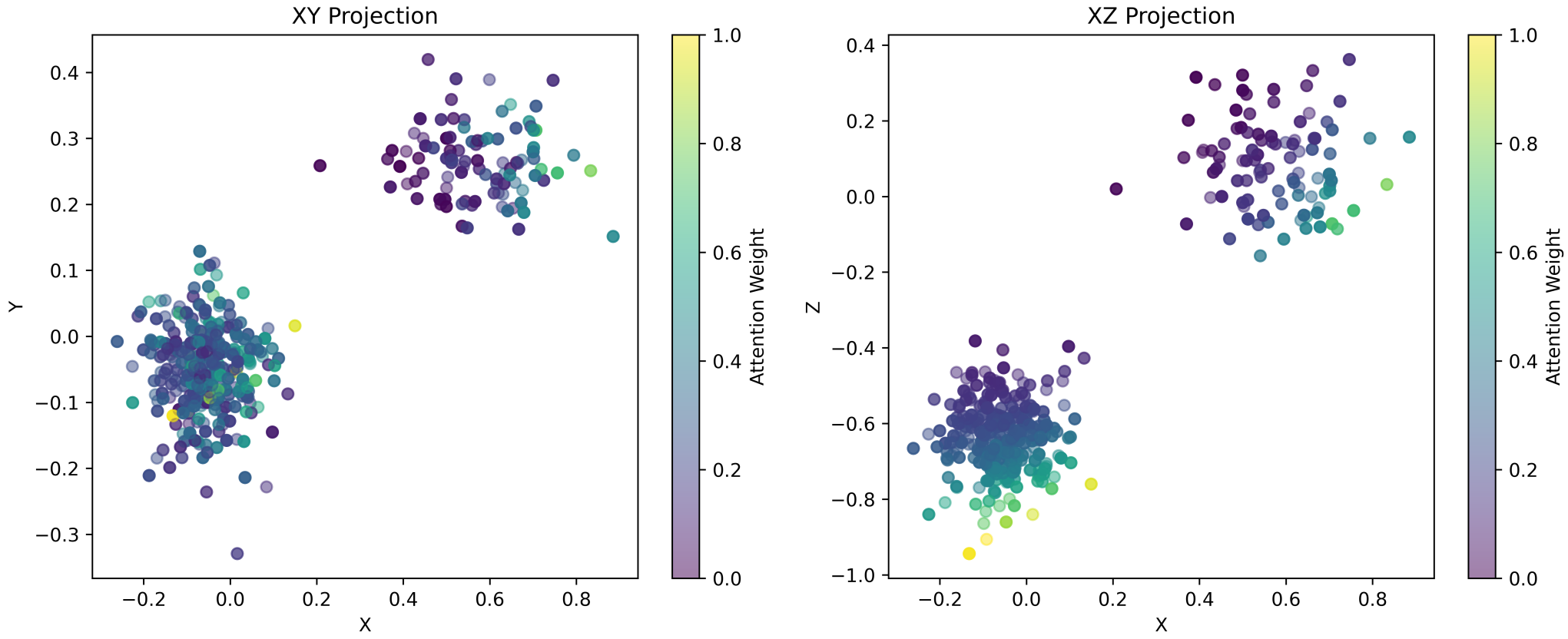

Figure S4 Visualization of the spatial attention for a tetrahedron with two clusters removed highlights the network's feature selection strategy. The attention map demonstrates a distinct focus on the peripheral regions of the point clusters, assigning significantly higher weights to points residing on the cluster exterior compared to the interior. This distribution indicates that the network learns to prioritize boundary topology as the most discriminative information for structural inference and reconstruction.

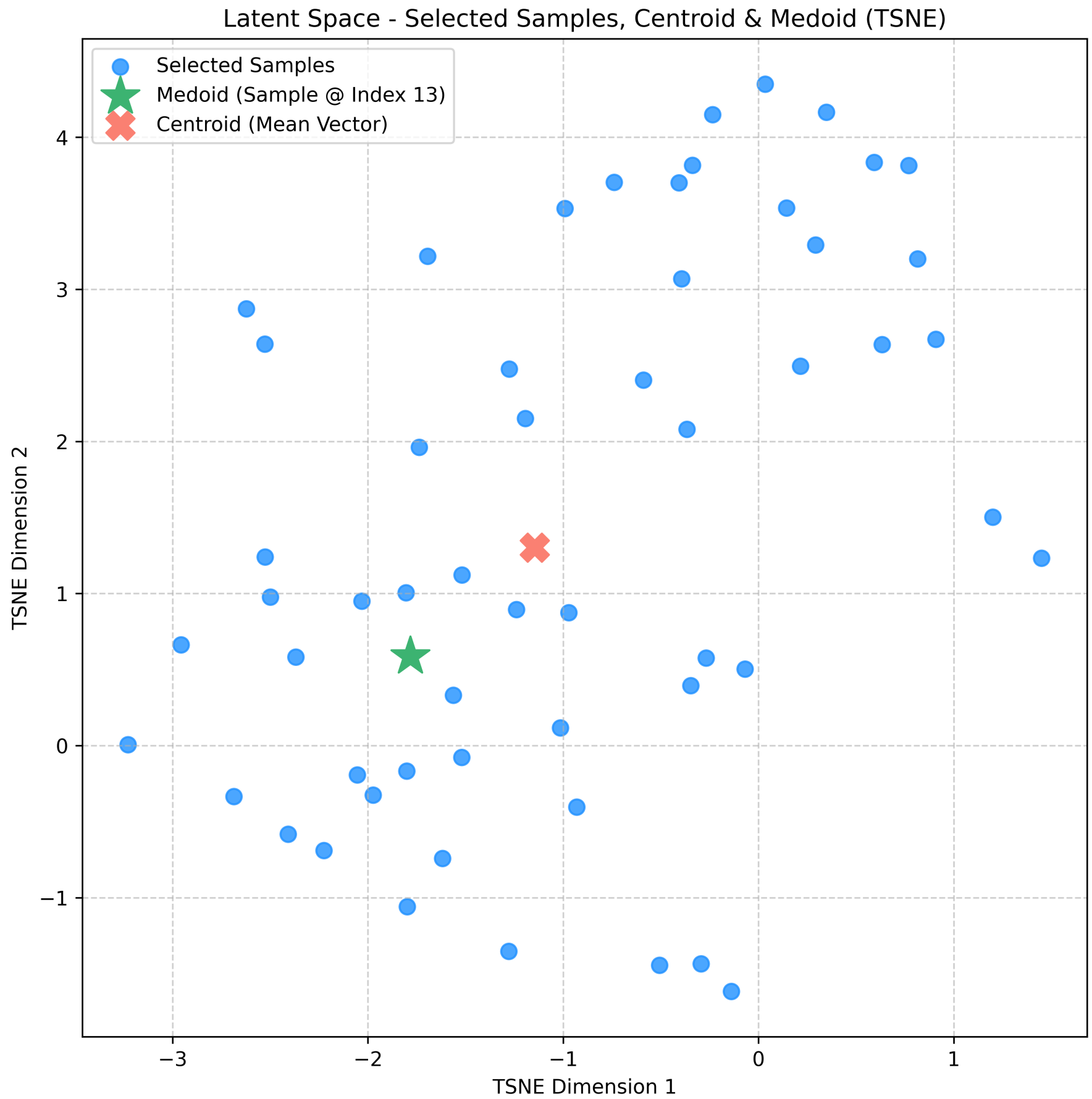

Figure S5 To generate a robust consensus structure, we compute the centroid (red cross), which represents the mathematical mean of all feature vectors in this cluster. However, to ensure the final reconstruction corresponds to a biologically valid conformation within the learned manifold, we identify the medoid (green star)—the specific experimental sample whose feature vector is closest to the centroid in terms of squared Euclidean distance. This medoid serves as the representative 'average' particle for subsequent decoding.

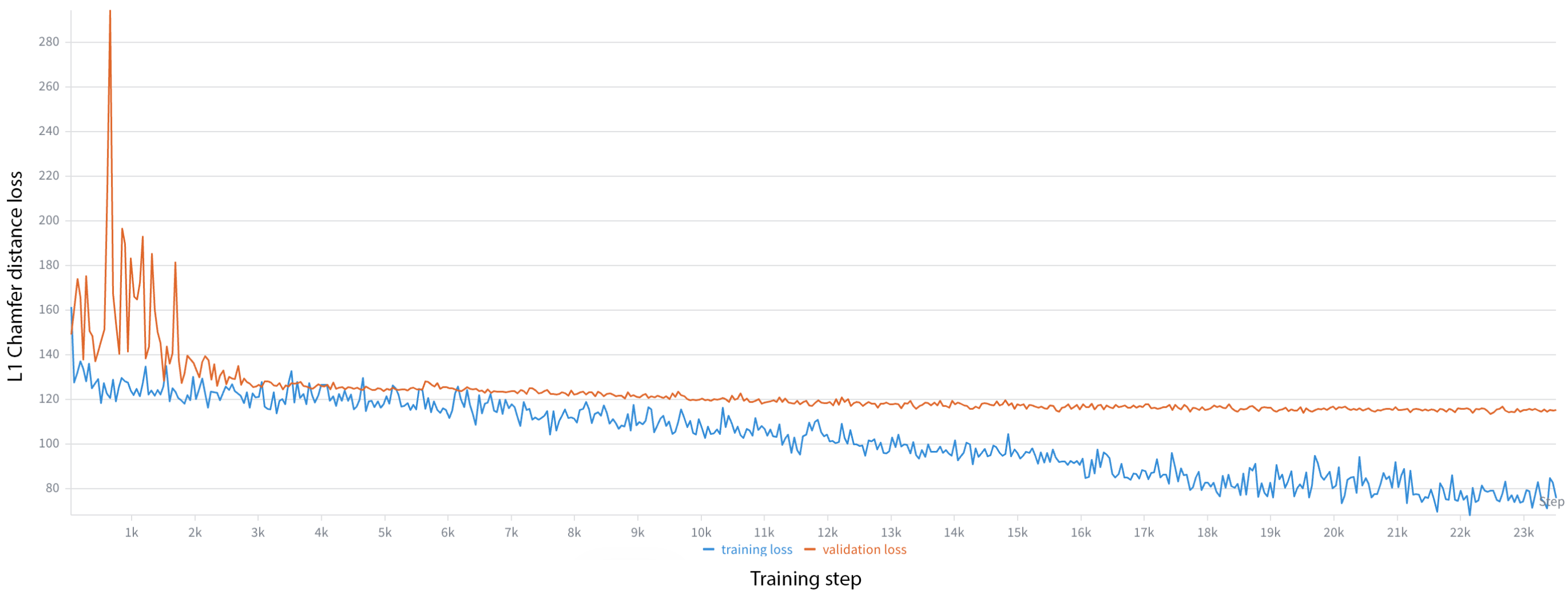

Figure S6 Training and validation loss during model optimization. The L1 Chamfer distance decreases steadily for the training set. The validation loss exhibits high initial volatility followed by a rapid early reduction, ultimately plateauing after roughly 5k steps.

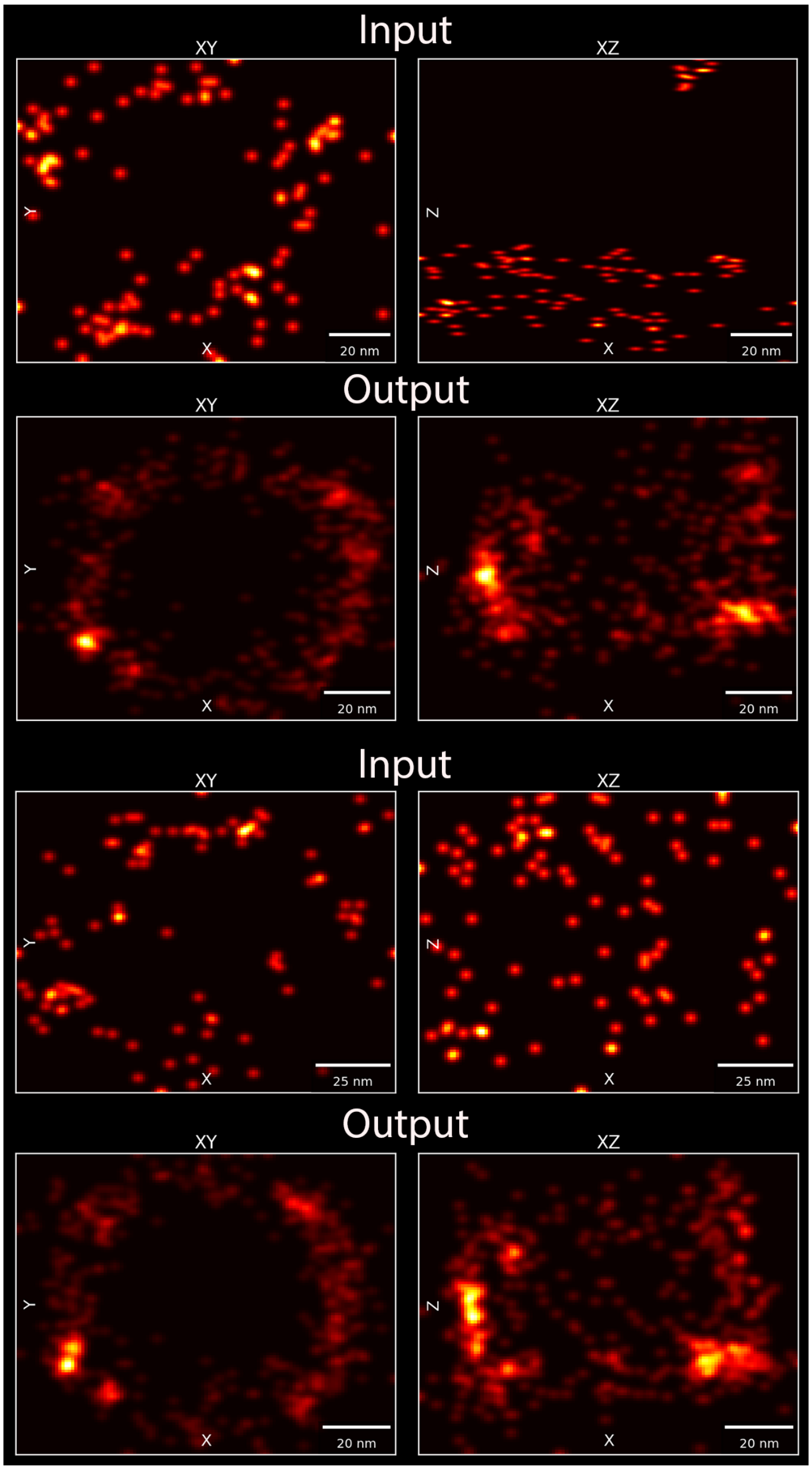

Figure S7 Outlier samples selected from the t-SNE feature-space visualization. Two samples identified as outliers in the t-SNE plot are shown with their corresponding network reconstructions. For each sample, the input point distribution and reconstructed output are visualized in XY and XZ projections. While the reconstructions recover the overall ring-like organization in the XY plane, the XZ projections show reduced axial fidelity: the two rings are not clearly separated along the z-axis, and reconstructed localizations are distributed throughout the inter-ring region.

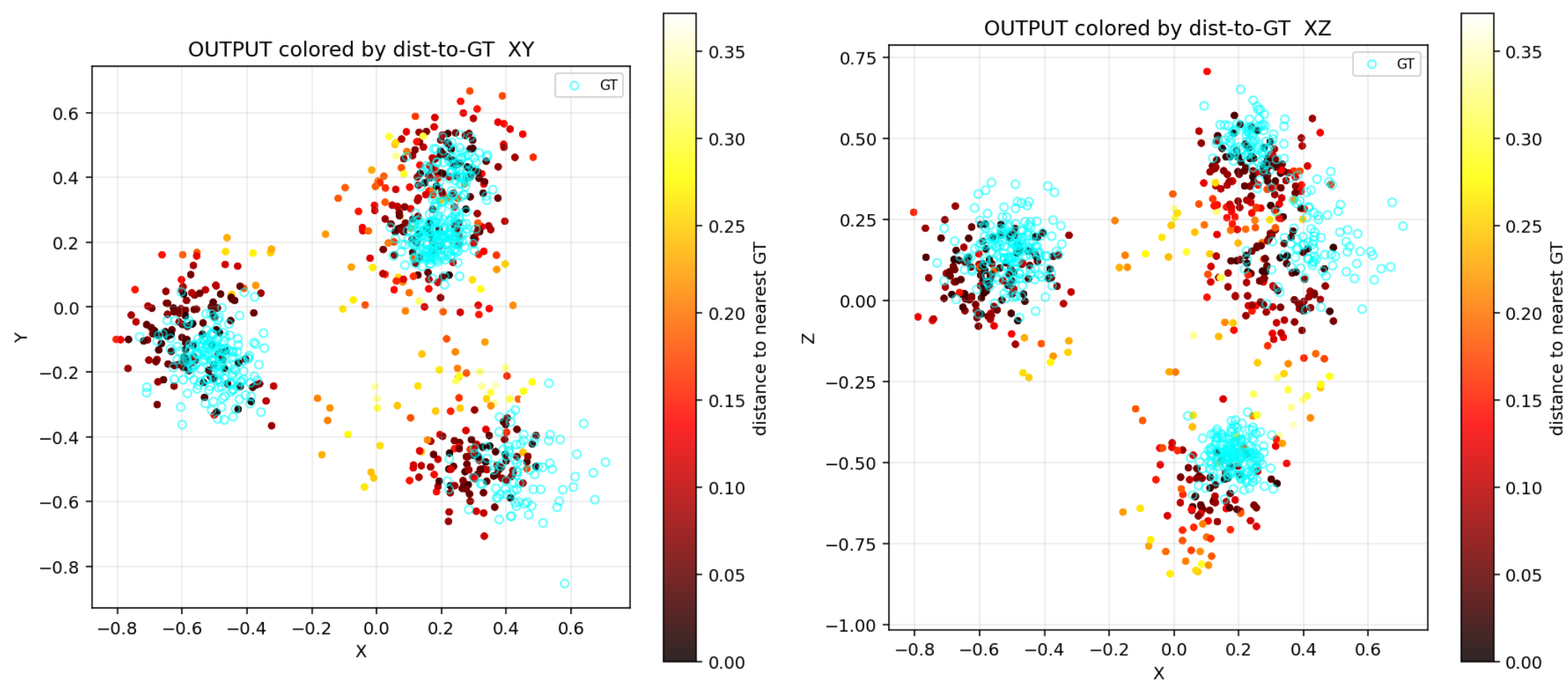

Figure S8 The reconstructed output is shown in XY and XZ projections and colored according to the distance of each reconstructed localization to its nearest ground-truth localization. Cyan circles indicate the ground-truth structure. Darker points correspond to smaller reconstruction errors, whereas yellow/white points indicate larger deviations. The error map shows that most reconstructed localizations remain close to the ground-truth rings, while higher-error points are mainly located in sparse regions between structural clusters and at the periphery of the reconstruction.

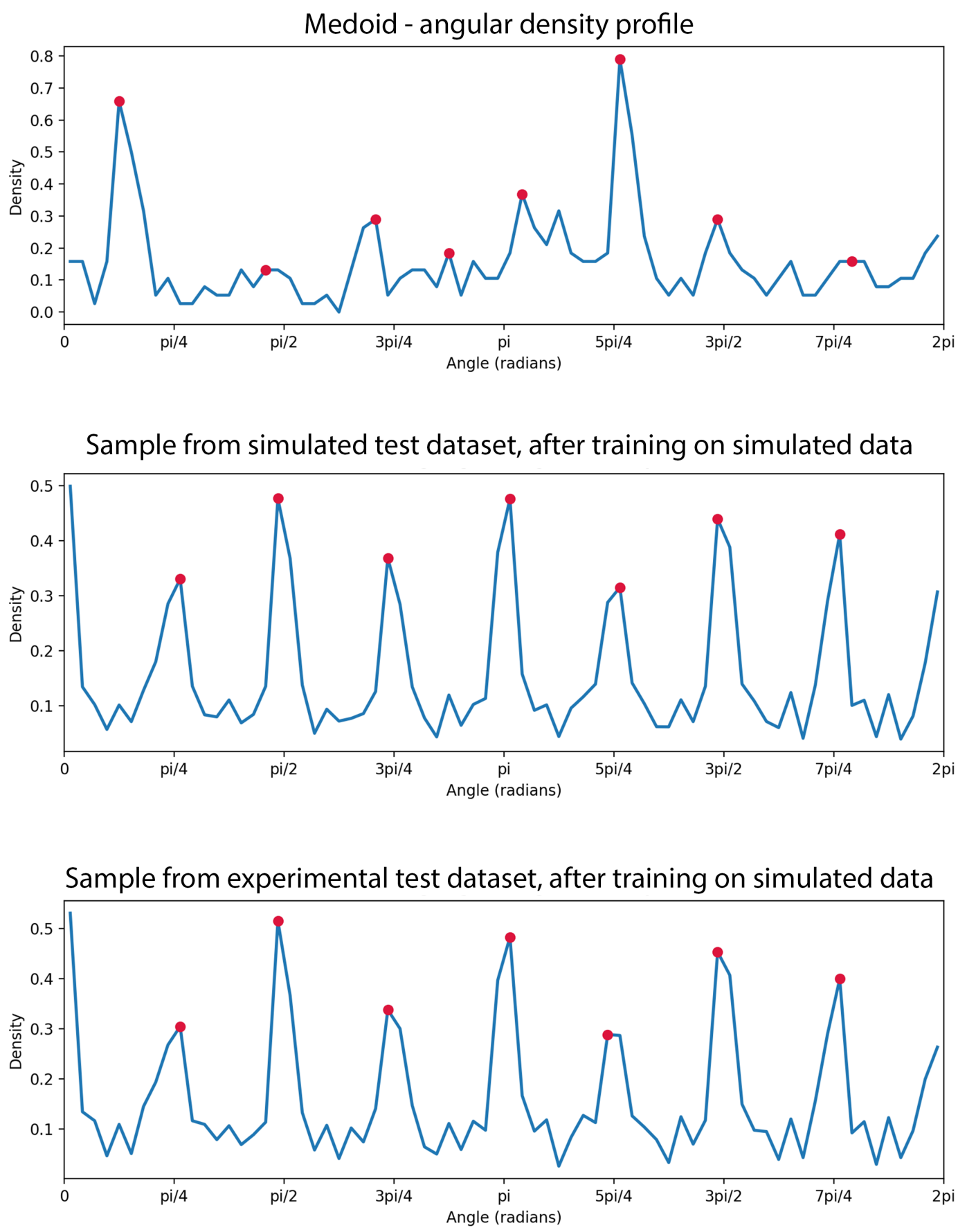

Figure S9 Angular density profiles of reconstructed Nuclear Pore Complex rings demonstrate the data-driven recovery of eight-fold rotational symmetry across simulated and experimental datasets. (Top) Angular density profile of the reconstructed experimental medoid. The distribution exhibits periodic modulations with approximately eight distinct density maxima (red dots). The observed variance in peak amplitudes and slight angular deviations reflects the model's preservation of authentic SMLM data stochasticity—including incomplete fluorophore labeling, linkage flexibility, and native conformational heterogeneity. (Middle) Profile of a reconstructed sample from the simulated test dataset. The distribution displays a strong, highly uniform periodicity with distinct maxima spaced at precisely $\pi/4$ (45-degree) intervals, confirming the network's algorithmic capacity to accurately learn the native octagonal architecture from ground-truth data. (Bottom) Profile of a sample from the experimental test dataset evaluated using the simulation-trained model. The successful recovery of the eight-fold periodic maxima demonstrates that the structural prior learned during simulation training generalizes to extract the underlying biological symmetry from noisy, incomplete experimental localizations.

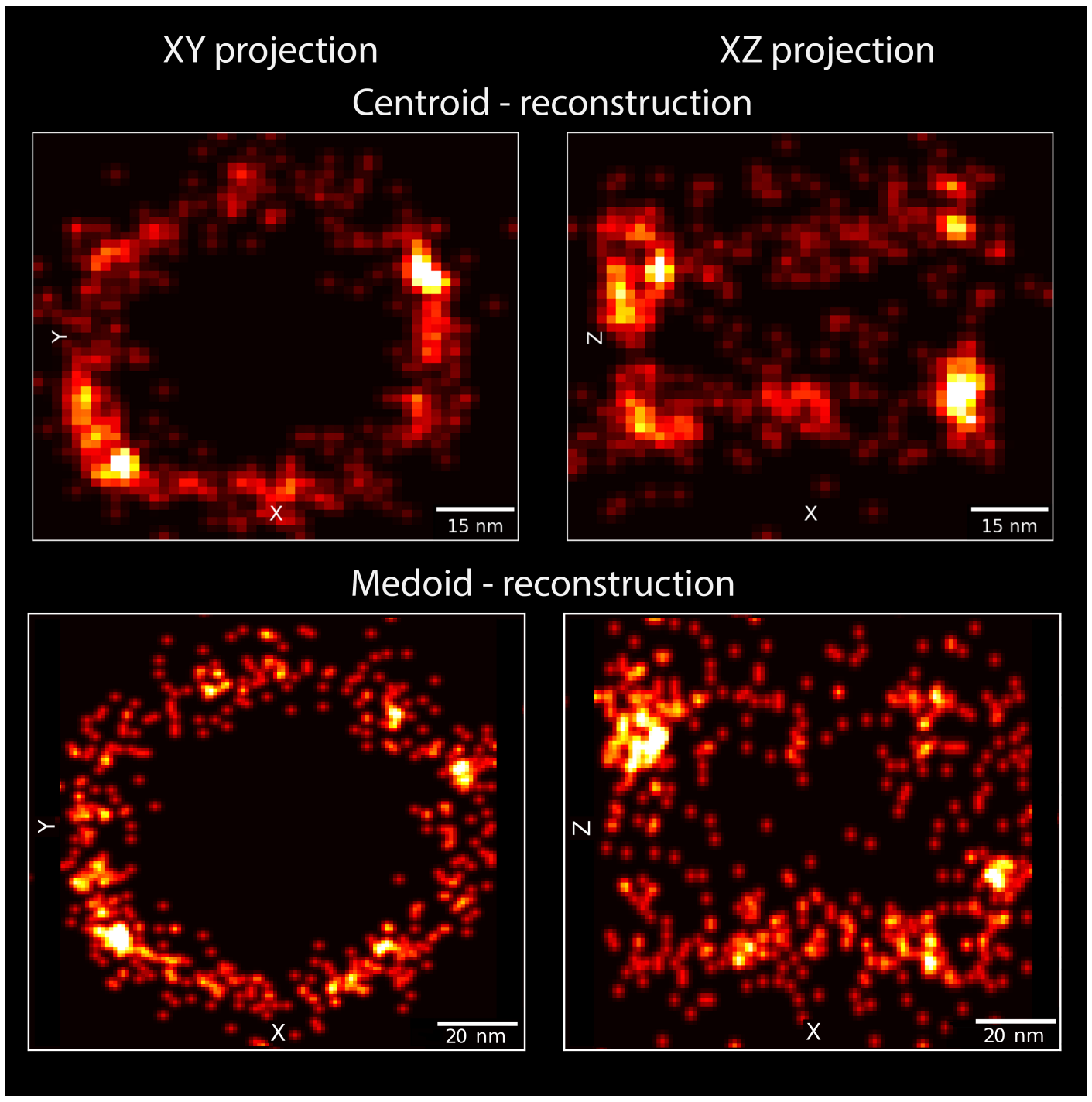

Figure S10 Structural comparison between centroid- and medoid-based reconstructions of the Nuclear Pore Complex. While the medoid represents a true experimental sample that preserves expected biological dimensions, the theoretically averaged centroid produces a structure with reduced geometric parameters (smaller ring radii and inter-ring distance). This dimensional shrinkage highlights that simple latent space averaging may produce out-of-distribution artifacts, suggesting that more sophisticated aggregation approaches would be necessary

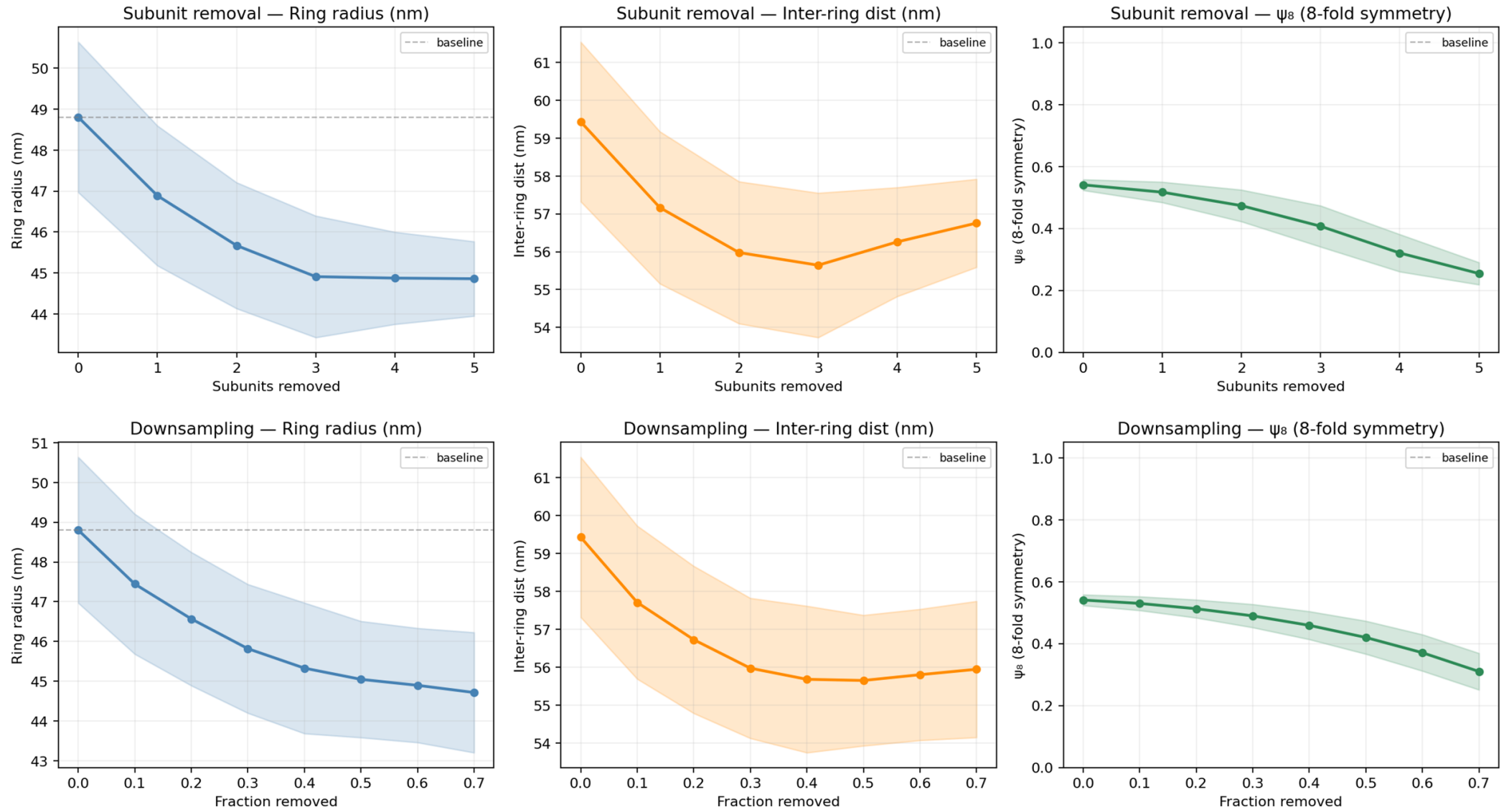

Figure S11 Performance evaluation of the Pocafoldas framework under systematic structural degradation and subunit removal. This series of reconstructions illustrates the impact of increasing data sparsity on the recovery of the canonical NPC architecture. Starting from a ground-truth reference with full eight-fold symmetry (top left), structural subunits were systematically removed alongside random localization downsampling to simulate extreme cases of incomplete labeling. As the number of missing subunits increases from one to seven, the reconstructed consensus structures (medoids) exhibit a quantifiable decline in geometric accuracy. This degradation is characterized by a decrease in the fidelity of the recovered ring radius and inter-ring distance compared to the known ground-truth dimensions. Furthermore, the quantitative recovery of the eight-fold symmetry, assessed via the Ψ_8 symmetry score, shows a progressive decrease as the input point clouds become increasingly sparse. While the model leverages learned structural priors to infer global topology from incomplete data, the precision of the final reconstruction remains constrained by a minimum threshold of available information.
